# A Comparative Evaluation of Surface Hardness and Nanomechanical Properties of Nickel-Titanium Orthodontic Wires: An In Vitro Study

**DOI:** 10.7759/cureus.64284

**Published:** 2024-07-10

**Authors:** Prasanth Prathapan Santhakumari, Vighnesh Varma Raja, Biju Kalarickal, Mohamed Haris Thavalam Parambil, Joseph Sebastian, Jacob Cheeramelil

**Affiliations:** 1 Department of Orthodontics and Dentofacial Orthopedics, Indira Gandhi Institute of Dental Sciences, Kothamangalam, IND; 2 Department of Orthodontics and Dentofacial Orthopedics, Annoor Dental College & Hospital, Muvattupuzha, IND; 3 Department of Orthodontics and Dentofacial Orthopedics, Mar Baselios Dental College, Kothamangalam, IND; 4 Department of Orthodontics and Dentofacial Orthopedics, Educare Institute of Dental Sciences, Malappuram, IND

**Keywords:** surface hardness, niti wires, nanoindentation, nanohardness, coated archwires

## Abstract

Objective: The study aimed to evaluate the influence of various surface coatings (epoxy, Teflon, and rhodium) on the surface roughness (SR) and nanomechanical characteristics of nickel-titanium (NiTi) archwires. The study compared these coated archwires to uncoated ones from a single manufacturer, which served as a control.

Materials and methods: There were 15 rectangular samples of four distinct archwires measuring 0.17 × 0.25. These were ultrasonically treated with an alkaline solution at 60°C for 15 minutes before being rinsed with distilled water to remove precipitates. With an orthodontic soft wire cutter, the straight buccal sections of coated and uncoated archwires were cut into 20 mm lengths. A three-dimensional optical noncontact surface profilometer evaluated the surface. Profilometers use contact scanning white light interferometry. Using the Vision64 software (Bruker Corporation, San Jose, CA), the profilometer's nanolens atomic force microscopy module has a completely automated turret with programmed X, Y, and Z motions. Images were taken in five random locations. Five average measurements matched specimen SR. A nanoindenter with a Berkovich diamond indenter measured nanohardness (NH) and elastic modulus (EM). The experimental results were analyzed using the Statistical Package for the Social Sciences software version 26.0 (SPSS Inc., Chicago, IL). To examine mean differences at 5% significance, analysis of variance and Tukey's post hoc test were applied for SR, NH, and EM.

Results: Wires coated with epoxy had the highest SR (1.499 ± 0.082), followed by Teflon (0.811 ± 0.023) and rhodium (0.308 ± 0.024). The SR of the control group was 0.289 ± 0.027. Significant differences in SR were found (p < 0.0001). Except for the comparison between rhodium and the control group (p = 0.684), all intergroup comparisons of SR showed significant differences (p < 0.0001). The rhodium-coated wires exhibited the highest NH (0.185 ± 0.014), and the epoxy group had the lowest (0.147 ± 0.017). Variations in NH were significant between the study groups (p < 0.0001). The epoxy, Teflon, and rhodium groups showed significant differences against the control group (p < 0.0001) in intergroup comparisons for NH. The Teflon group had the highest EM (5.367 ± 0.379), and the epoxy group had the lowest (5.012 ± 0.498). The EM of the control group was 56.946 ± 0.737. Results indicate considerable EM changes between the groups (p < 0.05). Comparisons between experimental and control groups showed significant differences (p < 0.0001).

Conclusion: The study's findings indicate that the SR of rhodium-coated archwires is substantially comparable to that of uncoated archwires. However, Teflon-, rhodium-, and epoxy-coated archwires had significantly different NH and EM compared to uncoated ones. Further, uncoated archwires have higher NH and EM.

## Introduction

The high need for improved esthetics during orthodontic treatment has prompted manufacturers to design appliances that blend satisfactory esthetics with suitable technical efficiency [1]. Manufacturing technologies have been established to enhance the visual appeal of orthodontic archwires (OAW), rendering them less visible while maintaining adequate color durability and clinical efficacy. Metallic OAW coated with inorganic or polymeric components is currently the only esthetically pleasing answer to this challenge [2]. Various claims have been made in the literature regarding esthetic-coated OAW. A study concerning coating adhesion and sliding qualities found that the coating reduced friction between OAW and brackets [3]. Few studies have reported issues with these coated OAWs, saying that OAWs lack sufficient color durability and that the coating film splits, exposing the underneath layer of the metal [4].

The OAW surface quality impacts the contact surface area and the esthetic consequence, corrosion behavior, and biological compatibility [2]. Given these issues with the coating, these wires remain commercialized and utilized in clinical settings, demonstrating the demand for cosmetic archwires with structural properties at least comparable to ordinary stainless steel and nickel-titanium(NiTi) archwires [5]. The OAW surface roughness (SR) impacts tooth movement efficacy, corrosion, friction, biocompatibility, esthetics, hygiene, and color durability. Archwire corrosion and SR, as well as ion release in the oral cavity, are positively associated. Increased SR increases frictional forces by increasing the surface area of contact between the bracket and OAW. This can diminish orthodontic force by 50% or more, minimizing the quality of orthodontic treatment [6,7].

Elastic modulus (EM) indicates archwire stiffness, which affects strength, springiness, and range. This, coupled with other variables such as surface hardness and SR, will influence the forces of friction between the archwire and bracket, resulting in tooth movement [8]. The orthodontists mostly use wires made of four types of basic base metal alloys: stainless steel, cobalt-chromium-nickel, NiTi, and beta-titanium. A range of tension and bending assessments were undertaken to measure yield strength and EM [9]. Recent improvements in nanoindentation testing have enabled the evaluation of mechanical characteristics in exceptionally tiny volumes of materials with a contact radius of less than 100 nm. The load and displacement evaluation of nanoindentation is tracked with a high-resolution displacement device throughout the indentation procedure, and the extent of indentation is determined from the geometric configuration of the indenter. Thus, the specimen's nanohardness (NH) can be estimated. Furthermore, using appropriate tools, the EM can be calculated theoretically from the load-displacement curve [10]. Suppose OAWs are to give predictable stresses to the teeth, clinicians must be cognizant of any effect that exposure to clinical conditions may have on both the mechanical and physical characteristics of the archwires. The current investigation sought to assess the influence of various surface coatings (epoxy-coated OAW, Teflon-coated OAW, and rhodium-coated OAW) on the SR and nanomechanical characteristics of NiTi OAWs. This was compared with uncoated wire from a single manufacturer, which was used as a control.

## Materials and methods

The sample size of the in vitro study was determined using G-Power version 3.1.9.7. (Heinrich Heine-Universität Düsseldorf, Düsseldorf, Germany) according to the results of a previously published study (effect size of 0.45 and power 80%) for SR measurement [11]. The sample consisted of 15 specimens of four distinct wires. Four different kinds of NiTi OAWs are as follows: epoxy-coated OAW, Teflon-coated OAW, rhodium-coated OAW, and uncoated OAW), assuming that all wires produced by a single manufacturer go through comparable processes for finishing [12]. The current investigation compared three distinct brands of coated esthetic and uncoated NiTi OAWs. The archwires were rectangular and had the same dimension (0.17 × 0.25). They were initially ultrasonically cleansed with an alkaline solution at 60°C for 15 minutes before being washed with distilled water to eliminate the precipitates.

Assessment of SR

Preparing and Testing Specimens

The specimens (n = 15 in each group) were newly packaged and preformed in arch forms. Each 20-mm length specimen was cut from the straight buccal sections of the esthetic-coated and uncoated OAWs with an OAW cutter. Distilled water was used to clean the surface contaminants of the cut wire. The cleansed wire was dried with tissue paper and stored for profilometry measurement. The surface was evaluated using a noncontact surface profilometer equipped with a three-dimensional optical trait (Bruker ContourGT, Tucson, AZ). The profilometer operates using contact scanning white light interferometry. The profilometer employs a nanolens atomic force microscopy (AFM) module. It features a completely automated turret with programmed X, Y, and Z motions powered by the Vision64 software (Bruker Corporation, San Jose, CA). The Vision64 software converts data with high resolution into precise three-dimensional visualizations. The OAW samples were fastened to the moveable turret, with the 0.025 surface pointing to the profilometer's illumination source. These samples were imaged in five random locations. The five mean measurements matched the specimen's SR (roughness average).

Assessment of nanomechanical characteristics

Preparing and Testing Specimens

The cleaned and dried OAWs were subjected to nanoindentation. The nanomechanical characteristics (NH and EM) were determined utilizing a nanoindenter (Bruker, Tucson, AZ, USA) with a Berkovich diamond indenter. The OAW samples were attached to the moveable turret with the 0.025 surface facing the indenter. The experiment was conducted in a confined chamber at 23°C with minimal noise levels. The indenter loading and unloading velocities were 0.01 and 0.02 mN/s, respectively. The resting time was five seconds at maximum load, with the load ranging from 1 to 10 mN. Every specimen received three random values, and the software attached to the nanoindenter instantaneously computed the average NH results. After determining the sample's hardness, the EM was calculated using a load-displacement curve. The EM was determined without the need for an additional test.

Statistical analysis

The results were analyzed using the Statistical Package for the Social Sciences software version 26.0 (SPSS Inc., Chicago, IL). SR, NH, and EM were determined utilizing analysis of variance and Tukey's post hoc multiple comparison tests to contrast the mean differences at a 5% significance level.

## Results

Table 1 displays the descriptive statistics for SR, NH, and EM.

**Table 1:** Comparison of the difference in SR, NH, and EM of the various archwires SR: surface roughness; Ra µm: roughness average in micrometers; NH: nanohardness; GPa: gigapascal; EM: elastic modulus; SD: standard deviation

Archwires	SR (Ra µm)	NH (GPa)	EM (GPa)
	Mean ± SD	Mean ± SD	Mean ± SD
Epoxy	1.499 ± 0.082	0.147 ± 0.017	5.012 ± 0.498
Teflon	0.811 ± 0.023	0.173 ± 0.012	5.367 ± 0.379
Rhodium	0.308 ± 0.024	0.185 ± 0.014	5.031 ± 0.184
Control	0.289 ± 0.027	0.198 ± 3.347	56.946 ± 0.737
F-test	2,284.06	379.928	41,524.723
P value	<0.0001	<0.0001	<0.0001

The epoxy-coated OAWs had the greatest SR characteristic value (1.499 ± 0.082), followed by Teflon (0.811 ± 0.023) and rhodium (0.308 ± 0.024). The control group exhibited an SR of 0.289 ± 0.027, as shown in Table 1. The SR measurements exhibited statistically significant differences (p < 0.0001). The SR showed significant differences among intergroup comparisons (p < 0.0001), except for the comparison between the rhodium-coated OAWs and uncoated OAWs (p = 0.684) (Table 2).

**Table 2:** Post hoc multiple comparison analysis of mean difference SR, NH, and EM of the various archwires SR: surface roughness; Ra µm: roughness average in micrometers; HSD: honestly significant difference; NH: nanohardness; GPa: gigapascal; EM: elastic modulus

Variables	Epoxy vs Teflon	Epoxy vs rhodium	Epoxy vs controls	Teflon vs rhodium	Teflon vs controls	Rhodium vs controls
SR (Ra µm)						
Mean difference	0.69	1.19	1.21	0.5	0.52	0.02
Tukey’s HSD	57.8	100.09	101.66	42.29	43.86	1.57
P value	<0.0001	<0.0001	<0.0001	<0.0001	<0.0001	0.684
NH (GPa)						
Mean difference	0.03	0.04	3.2	0.01	3.17	3.16
Tukey’s HSD	1.01	1.49	123.92	0.49	122.91	122.43
P value	0.8919	0.7168	<0.0001	0.9857	<0.0001	<0.0001
EM (GPa)						
Mean difference	0.36	0.02	51.93	0.34	51.58	51.92
Tukey’s HSD	2.79	0.15	408.52	2.65	405.73	408.38
P value	0.209	0.999	<0.0001	0.252	<0.0001	<0.0001

Within the experimental groups, rhodium-coated wires exhibited the highest NH value (0.185 ± 0.014), whereas the epoxy group exhibited the lowest value (0.147 ± 0.017). The NH of the control group was measured to be 0.198 ± 3.347. The NH results exhibited statistically significant variations (p < 0.0001). Intergroup evaluation, however, showed statistically significant differences (p < 0.0001) between the epoxy-coated OAWs and uncoated OAW groups, Teflon and control groups, and rhodium and control groups (Table 2).

In comparison, the Teflon group demonstrated the highest EM values (5.367 ± 0.379), while epoxy had the lowest readings (5.012 ± 0.498). The EM of the control group was found to be 56.946 ± 0.737. The EM measurements indicated statistically significant variations (p < 0.05). The intergroup assessment revealed that the experimental and control groups differed statistically significantly (p < 0.0001) (Table 2).

## Discussion

The current study assessed the SR and nanomechanical characteristics of the epoxy-coated OAW, Teflon-coated OAW, and rhodium-coated OAWs, using uncoated wire from a single manufacturer to serve as a control. Historically, archwires have been evaluated for their mechanical and physical characteristics by distinct measurements conducted utilizing various procedures such as three-point bend assessments, tensile testing, and Rockwell and Vickers hardness measurements. SR has been evaluated through the utilization of surface profilometry, several optical techniques, and AFM. Nevertheless, it is feasible to concurrently evaluate all three features by employing an AFM in conjunction with a nanoindenter. Nanoindentation is a type of indentation testing where the extent of the indent is measured in nanometers. This method enables concurrent quantitative assessments of both hardness and EM. Additionally, when combined with an AFM, it also captures SR data [8]. The current study assessed the SR of the OAWs employing a noncontact profilometer, which is considered the best and most important method for examining the SR of the OAWs [13]. Nevertheless, only a few studies have expressed criticism regarding the application of profilometry for SR measurement, highlighting its failure to assess the entirety of the surface area [14].

This study found that esthetic archwires coated with epoxy had the highest SR measurements, whereas uncoated OAWs and rhodium-coated OAWs had the lowest measurements. Moreover, there was a notable disparity in SR measurements recorded among the esthetic-coated OAWs. The variation may be attributed to the distinct coating applied to the OAWs. This observation is consistent with the results of the earlier documented investigations [13,14]. The NH of the coated archwires in this investigation ranged from 0.147 ± 0.0168 to 0.185 ± 0.0136 GPa, while the EM varied from 5.367 ± 0.379 to 5.012 ± 0.498 GPa. No notable discrepancy was observed in the esthetic-coated OAWs when measuring the NH and EM. This finding aligns with the prior research of da Silva et al., in which they examined four esthetic-coated OAWs and reported comparable measurements of NH and EM [5]. This conclusion indicates that the various coatings on the archwires had minimal impact on the mechanical qualities. The aforementioned perspective was verified by Bącela et al., who examined two esthetic archwires. The study indicated that the coating technique, rather than the specific kind of coating, had a major impact on the mechanical characteristics and surface morphology of the archwires [7]. The notable difference observed among the archwires examined in this study highlights the importance of carefully choosing esthetic archwires to minimize the negative impacts produced by their rough surfaces.

There have been reports indicating that the mechanical characteristics of certain coated wires were notably inferior to those of uncoated wires [15]. The decline in the physical quality of wires has a negative impact on orthodontic therapy [16]. Coated esthetic archwires consist of a metallic wire core that is covered with a polymer coating that matches the color of teeth. The archwire can be coated with either organic [17] or inorganic components [18] to conceal the metal and give it a glossy appearance similar to enamel. Applying a coating enhances the visual appeal and alters the surface, potentially impacting the friction, corrosive characteristics, and mechanical resilience of the wires. The coated wires are frequently subject to damage caused by chewing and the activation of enzymes [19].

Previous investigations have revealed that the epoxy resin covering becomes unstable when exposed to saliva [17,20-22], thereby exhibiting a range of adverse effects, including heightened SR, tearing, and loss of coating in various areas. The instability, characterized by the undesirable discoloration and disintegration of the coating layer [23], may occur due to the hydrophilic nature of resins [24]. The hardness value provides information about the coating's ability to withstand scratching and plastic deformation [25]. The integrity and esthetics of the coating are better preserved when the coating layer is harder because it is more resistant to plastic deformation. Aboalnaga and Shahawi [26] found that the majority of coated archwires ultimately exhibited unfavorable surface alterations after being exposed to the intraoral milieu. The physical qualities of the coating, such as SR and hardness, impact these surface alterations. Additional research is necessary to assess the impact of SR and NH of the coated archwire on the resultant friction. Furthermore, it is necessary to conduct in vivo research that investigates the impact of the oral setting on these same characteristics.

Despite its strengths, the study has several limitations. The sample size was relatively small, with only 15 samples per group, which may limit the generalizability of the findings. Additionally, the study was conducted under controlled laboratory conditions, which may not fully replicate the complex environment of the oral cavity where various factors, such as saliva, temperature fluctuations, and mechanical stresses, could influence the performance of the archwires. The study also focused solely on surface coatings from a single manufacturer, which may not represent the full range of available commercial products. Long-term clinical trials involving a diverse patient population would provide valuable insights into the performance and durability of different surface-coated NiTi archwires in real-world conditions. Additionally, investigating the impact of other environmental factors present in the oral cavity on the mechanical properties of coated and uncoated archwires would offer a more comprehensive understanding of their clinical efficacy. Expanding the scope of the study to include coatings from multiple manufacturers could help determine if the observed effects are consistent across different products.

## Conclusions

The current investigation determined that rhodium-coated OAWs exhibited SR nearly identical to uncoated OAWs. However, all coated OAWs (epoxy, Teflon, and rhodium) displayed significant differences in NH and EM compared to uncoated OAWs. Specifically, uncoated archwires demonstrated higher NH and EM values than coated archwires. Epoxy-coated wires had the highest SR and lowest NH, while Teflon-coated wires had the highest EM. Overall, the study highlights that surface coatings on NiTi archwires can significantly affect their mechanical properties, with rhodium-coated wires being the most comparable to uncoated wires in terms of SR.
